# Nonlocal teleparallel cosmology

**DOI:** 10.1140/epjc/s10052-017-5210-1

**Published:** 2017-09-20

**Authors:** Sebastian Bahamonde, Salvatore Capozziello, Mir Faizal, Rafael C. Nunes

**Affiliations:** 1 Department of Mathematics, University College London, Gower Street, London, WC1E 6BT UK; 2 Dipartimento di Fisica “E. Pancini”, Universitá di Napoli “Federico II”, Naples, Italy; 3 Gran Sasso Science Institute, Via F. Crispi 7, 67100 L’Aquila, Italy; 4 INFN Sez. di Napoli, Compl. Univ. di Monte S. Angelo, Edificio G, Via Cinthia, 80126 Naples, Italy; 5 Irving K. Barber School of Arts and Sciences, University of British Columbia - Okanagan, 3333 University Way, Kelowna, BC V1V 1V7 Canada; 6 Department of Physics and Astronomy, University of Lethbridge, Lethbridge, AB T1K 3M4 Canada; 7 Departamento de Física, Universidade Federal de Juiz de Fora, Juiz de Fora, MG 36036-330 Brazil

## Abstract

Even though it is not possible to differentiate general relativity from teleparallel gravity using classical experiments, it could be possible to discriminate between them by quantum gravitational effects. These effects have motivated the introduction of nonlocal deformations of general relativity, and similar effects are also expected to occur in teleparallel gravity. Here, we study nonlocal deformations of teleparallel gravity along with its cosmological solutions. We observe that nonlocal teleparallel gravity (like nonlocal general relativity) is consistent with the present cosmological data obtained by SNe Ia + BAO + CC + $H_0$ observations. Along this track, future experiments probing nonlocal effects could be used to test whether general relativity or teleparallel gravity gives the most consistent picture of gravitational interaction.

## Introduction

General Relativity (GR) tells us that gravitational interaction is described by the curvature of torsion-less spacetimes. On the other hand, it is possible to describe gravity by the torsion of spacetime, so that the curvature picture is not necessary. A theory where gravity is described by the torsion of spacetime (without curvature) is called the Teleparallel Equivalent of General Relativity (TEGR) [1–4]. Even though these two approaches are fundamentally different, they produce the same classical field equations. Thus, both theories predict the same dynamics for classical gravitational systems, and so classical gravitational experiments cannot be used to test which of them gives the correct theory of gravity. In other words, they are equivalent at the classical level.

However, because these theories are conceptually different, they are expected to produce different quantum effects. An important remark is in order at this point. We can deal with TEGR only at the classical level because it produces the same classical field equations as GR. Considering quantum effects and nonlocality, it is improper to speak of equivalence of the two theories since they could be fundamentally different. Due to this fact, we will speak of teleparallel gravity in general and of TEGR in the classical case.

Even though we do not have a fully developed quantum theory of gravity, there are various proposals for quantum gravity, and a universal prediction from almost all of these approaches seem to be the existence of an intrinsic extended structure in the geometry of spacetime [5, 6], and such an extended structure would be related to an effective nonlocal behavior for spacetime [7–10]. For example, in perturbative string theory, it is not possible to measure spacetime below the string length scale, as the string is the smallest available probe. As it is not possible to define point-like local structures, string theory produces an effective nonlocal behavior [11, 12]. Similarly, there is an intrinsic minimal area in loop-quantum gravity [13], and this extended structure is expected to produce a nonlocal behavior. It can be argued, from black hole physics, that any theory of quantum gravity should present intrinsic extended structures of the order of the Planck length, and it would not be possible to probe the spacetime below this scale. In fact, the energy needed to probe the spacetime below this scale is more than the energy needed to form a mini black hole in that region of spacetime [14, 15].

Thus, quantum gravitational effects produce effective extended structures in spacetime that would lead to nonlocality [5, 6]. Hence, it can be argued that the first order corrections from quantum gravity will produce nonlocal deformations of GR [16–18], and this will, in turn, produce a nonlocality in cosmology. The effect of nonlocal deformations in cosmology could be a straightforward explanation for cosmic acceleration [19–22].

Furthermore, the nonlocality induced by GR deformations could be important to understand better the transition from radiation to matter dominated era if consistently constrained with the observations.

As nonlocality is produced by first order quantum gravitational effects, it is expected that they would also occur in teleparallel gravity. Unlike the standard local classical dynamics, the behavior of such nonlocal effects could be very different in teleparallel gravity and GR, and they can be used to experimentally discriminate between these two theories. Therefore, it is interesting to study the nonlocal deformation of both GR and teleparallel gravity. Even thought the nonlocal deformation of GR has been extensively studied, the nonlocal deformation of TEGR has not been studied. Thus, in this paper, we will analyze a model of nonlocal teleparallel gravity.

We will observe that at present, the nonlocal teleparallel gravity satisfies all the existing cosmological experimental constraints, and can explain phenomena that are explained using nonlocal deformations of GR. However, as the nonlocal teleparallel gravity is fundamentally different from nonlocal deformation of GR, future experiments can be used to verify which of these theories is the correct theory of gravity. Thus, the action for general relativity $\mathcal {S}_\mathrm{GR}$ can be corrected by a nonlocal term $\mathcal {S}_\mathrm{SRNL}$ due to quantum corrections, and so the quantum corrected nonlocal GR can be written as [19, 20]

1 $$\begin{aligned} {\mathcal {S}}_1 = {\mathcal {S}}_\mathrm{GR} + {\mathcal {S}}_\mathrm{GRNL}. \end{aligned}$$

Similarly, the standard classical action of TEGR $\mathcal {S}_\mathrm{TEGR}$ can be corrected by a nonlocal term due to quantum corrections $\mathcal {S}_\mathrm{TEGRNL}$, and so the quantum corrected nonlocal teleparallel gravity can be written as

2 $$\begin{aligned} \mathcal {S}_2 = \mathcal {S}_\mathrm{TEGR} + \mathcal {S}_\mathrm{TEGRNL}. \end{aligned}$$

It is not possible to experimentally differentiate between $\mathcal {S}_\mathrm{GR}$ and $\mathcal {S}_\mathrm{TEGR}$, but the quantum corrections to these theories $\mathcal {S}_\mathrm{GRNL}$ and $\mathcal {S}_\mathrm{TEGRNL}$ are very different. Thus, it is experimentally possible to discriminate between $\mathcal {S}_1$ and $\mathcal {S}_2$. It may be noted that like in nonlocal GR case, the nonlocal correction to teleparallel gravity is motivated by quantum gravitational effects, and it is not arbitrary added to the original action.

It may be noted that nonlocal teleparallel formalism could be a better approach to the study of quantum gravitational effects. This is due to the fact that TEGR does not require the equivalence principle to be formulated (see Chapter 9 in [2]), and it has been argued that quantum effects can cause the violation of the equivalence principle [24]. Furthermore, a violation of the equivalence principle can be related to a violation of the Lorentz symmetry [25], and Lorentz symmetry is also expected to be break at the UV scale in various approaches to quantum gravity, such as discrete spacetime [26], spacetime foam [27], spin-network in loop-quantum gravity (LQG) [28], non-commutative geometry [29, 30], ghost condensation [31] and Horava–Lifshitz gravity [32, 33]. In teleparallel theories of gravity, there are two different approaches. The first one does not assume that the spin connection (which is related to inertial effects) is zero, making all the quantities invariant under Lorentz transformations. This formalism was implemented firstly in modifications of teleparallel theories of gravity in *f*(*T*) gravity in [34]. The second approach is the one where a specific frame is chosen at the beginning of the theory, or in other words, where one chooses the spin connection equal to zero. When Einstein and later Weitzenböck formulated the teleparallel equivalent of general relativity theory, they chose that formalism. This approach is sometimes called the “pure tetrad” formalism or the “Weitzenböck gauge” teleparallel formalism. In this formalism, the torsion tensor does not transform covariantly under local Lorentz transformations. Hence, the torsion scalar also is not invariant under local Lorentz transformations. In standard teleparallel gravity where just a linear combination of the scalar torsion is considered in the action $\mathcal {S}_\mathrm{TEGR}$, the theory becomes quasi-local Lorentz invariant, or invariant up to a boundary term. However, when one is considering modifications of teleparallel theories of gravity, such as *f*(*T*) gravity or in our case nonlocal teleparallel gravity, the theory is no longer local Lorentz invariant. In that case, in terms of computations, one way to alleviate this issue is by introducing the so-called “good tetrad” [35]. Mostly all the papers related to *f*(*T*) gravity work with this formalism so that in this work, we will follow it (see [36]). Further, the lost of this invariance in teleparallel theories might be an interesting behavior on quantum scales. For a detailed analysis related to the covariance of teleparallel theories of gravity, see [34, 37–39].

In this paper, we will study a nonlocal deformation of Teleparallel Gravity, and the nonlocal cosmological solutions obtained from such a deformed theory. Furthermore, we propose a way to experimentally discriminate teleparallel gravity from GR at quantum scales. The paper is organized as follows. In Sect. 2, we discuss the action and the field equations of nonlocal teleparallel Gravity. Observational constrains coming from cosmology are given in Sect. 3. These constraints result useful discriminate between nonlocal GR and nonlocal teleparallel gravity. Conclusions are drawn in Sect. 4. Appendix A is devoted to details of the derivation of the field equations.

## Nonlocal teleparallel gravity 

In this section, we will obtain a nonlocal deformation of teleparallel gravity. Adopting the formalism developed for nonlocal deformations of GR [19, 20], we can write a nonlocal deformation for teleparallel gravity as

3 $$\begin{aligned} \mathcal {S}&=\frac{1}{2\kappa }\int \mathrm{d}^{4}x\, e(x) \, T(x)\left[ f(\mathcal {G}[T](x))-1 \right] +\int \mathrm{d}^{4}x\, e(x)\,L_{m} \end{aligned}$$

4 $$\begin{aligned}&=\mathcal {S}_\mathrm{TEGR}+\frac{1}{2\kappa }\int \mathrm{d}^{4}x\, e(x) \, T(x)f\left( (\square ^{-1}T)(x) \right) \nonumber \\&\quad +\int \mathrm{d}^{4}x\, e(x)\,L_{m}, \end{aligned}$$

where $\kappa =8\pi G$, *T* is the torsion scalar, $e=\text {det}(e^{a}_{\mu })=\sqrt{-g}$, *f* is an arbitrary function which depends on the retarded Green function evaluated at the torsion scalar (quantum effects such as the Planck constant have been absorbed in the definition of this function), $L_{m}$ is any matter Lagrangian, $\square \equiv \partial _{\rho }(e g^{\sigma \rho }\partial _{\sigma })/e$ is the scalar-wave operator, and $\mathcal {G}[f](x)$ is a nonlocal operator which can be written in terms of the Green function $G(x,x')$ as

5 $$\begin{aligned} \mathcal {G}[f](x)= & {} (\square ^{-1}f)(x)\nonumber \\= & {} \int \mathrm{d}^4x'\, e(x') f(x')G(x,x'). \end{aligned}$$

Furthermore, like the nonlocal corrections to the GR, these nonlocal corrections to the teleparallel gravity are also motivated from quantum gravitational effects. We note that, as for nonlocal GR, the Green function is evaluated at the Ricci scalar *R*, in nonlocal teleparallel gravity, the Green function is evaluated at the torsion scalar *T* (for the sake of simplicity, we write *T*(*x*) as *T* and *e*(*x*) as *e*).

It is worth noticing that (unlike GR, which produces the same equations of motion as TEGR), the nonlocal deformation of GR is different from the nonlocal deformation of teleparallel gravity. The latter comes from the fact that $R=-T+B$, where *B* is a boundary term so that $\mathcal {S}_{GR}$ (which is constructed by *R*) and $\mathcal {S}_{TEGR}$(which is constructed by *T*) produces the same field equations. However, the nonlocal terms $\sqrt{-g}Rf_1(\Box ^{-1}R)$ and $eTf_2(\Box ^{-1}T)$ coming from the nonlocal actions $\mathcal {S}_\mathrm{GRNL}$ and $\mathcal {S}_\mathrm{TEGRNL}$ will produce different field equations even for the case where $f_1=\Box ^{-1}R$ and $f_2=\Box ^{-1}T$. This happens since the boundary term *B*, which is the difference between *T* and *R*, produces a contribution in the variational process in nonlocal terms. This fact is in the same spirit as the discussion in [38, 40], where it was shown that *f*(*R*) and *f*(*T*) gravity (generalizations of $\mathcal {S}_\mathrm{GR}$ and $\mathcal {S}_\mathrm{TEGR}$, respectively), are different for this boundary term and the way to connect these two theories is to consider a more general action where the function depends on both the boundary term and the scalar torsion, the so-called *f*(*T*, *B*) gravity (see also [41, 42]). Moreover, the same happens when one considers more general theories like modified Gauss–Bonnet *f*(*R*, *G*) gravity [43] and teleparallel modified Gauss–Bonnet gravity $f(T,T_{G})$ [44] where two boundary terms $f(T,B,T_{G},B_{G})$ need to be taken into account in order to connect the two theories (for more details, see [45]). Similarly, it is also possible to construct a general scalar tensor theory by considering non-minimally couplings between the scalar field and both the scalar torsion and the boundary term (see [46, 47]). By doing that, one can also recover other well-known scalar tensor theories such as quintessence or non-minimally coupled curvature–scalar field theory. Exactly as in those cases, in principle, one can extend the action (3) changing $eTf(\Box ^{-1}T)$ by $e f_1(T,B)f_2(\Box ^{-1}T,\Box ^{-1}B)$ and hence we achieve a more general theory which can connect nonlocal teleparallel gravity with nonlocal GR for the cases $f_{1}=-T+B$ and $f_2=-\Box ^{-1}T+\Box ^{-1}B$ [48].

By a variation with respect to the tetrad, we obtain

6 $$\begin{aligned} \delta \mathcal {S}= & {} \delta \mathcal {S}_\mathrm{TEGR}+\frac{1}{2\kappa }\int \left[ T f(\mathcal {G}[T])\delta e+e f(\mathcal {G}[T])\delta T \right. \nonumber \\&\left. +\, e\, T\delta f(\mathcal {G}[T]) \right] \mathrm{d}^{4}x+\int \mathrm{d}^{4}x\delta (eL_{m}), \end{aligned}$$

where

7 $$\begin{aligned} e f(\mathcal {G}[T]) \delta T= & {} -4 \left[ e(\partial _{\mu }f(\mathcal {G}[T]))S_{a}\,^{\mu \beta }+\partial _{\mu }(e S_{a}\,^{\mu \beta })f(\mathcal {G}[T])\right. \nonumber \\&\left. -ef(\mathcal {G}[T])T^{\sigma }\,_{\mu a}S_{\sigma }\,^{\beta \mu } \right] \delta e^{a}_{\beta }, \end{aligned}$$

8 $$\begin{aligned} T f(\mathcal {G}[T]) \delta e = eTf(\mathcal {G}[T]) E_{a}^{\beta } \delta e^{a}_{\beta } \,, \end{aligned}$$

9 $$\begin{aligned} eT\delta f(\mathcal {G}[T])= & {} e\Big [T\mathcal {G}[Tf'(\mathcal {G})]E_{a}^{\beta }+\partial _{\mu }(\mathcal {G}[Tf'(\mathcal {G})])(\partial _{\nu }T)\nonumber \\&\times \Big (g^{\mu \nu }E_{a}^{\beta }-2g^{\beta (\mu }E_{a}^{\nu )}\Big )\Big ]\delta e^{a}_{\beta }\nonumber \\&+e\,\mathcal {G}[Tf'(\mathcal {G})]\delta T\,. \end{aligned}$$

See Appendix 1 for details of the variation of the nonlocal term (9). It is worth noticing that the energy-momentum tensor is

10 $$\begin{aligned} \Theta ^{\beta }_{a}=e^{-1} [{\delta (eL_{m})}/{\delta e^{a}_{\beta }}], \end{aligned}$$

so the field equations for Nonlocal teleparallel gravity can be written as

11 $$\begin{aligned}&4\Big [ S_{a}\,^{\mu \beta }\partial _{\mu }+\frac{1}{e}\partial _{\mu }(e S_{a}\,^{\mu \beta })-T^{\sigma }\,_{\mu a}S_{\sigma }\,^{\beta \mu } -T\,E_{a}^{\beta }\Big ]\nonumber \\&\quad \times \Big [f(\mathcal {G}[T])+\mathcal {G}[Tf'(\mathcal {G})]\Big ]\nonumber \\&\quad -\frac{4}{e}\partial _{\mu }(e S_{a}\,^{\mu \beta })+4T^{\sigma }\,_{\mu a}S_{\sigma }\,^{\beta \mu }+TE_{a}^{\beta }-\partial _{\rho }\nonumber \\&\quad \times \Big (\mathcal {G}[Tf'(\mathcal {G})]\Big )(\partial _{\sigma }T)\Big (g^{\sigma \rho }E_{a}^{\beta }-2g^{\beta (\rho }E_{a}^{\sigma )}\Big )=2\kappa \Theta _{a}^{\beta }.\nonumber \\ \end{aligned}$$

We have obtained the field equations for the nonlocal deformation of teleparallel gravity, and now we will analyze a nonlocal cosmological solution coming from this nonlocal model of gravity.

Let us assume a Friedman–Lemaître–Robertson–Walker (FLRW) cosmology with the following tetrad in Euclidean coordinates $$ e^{a}_{\beta }=(1,a(t),a(t),a(t)),$$ and write the FLRW metric as $$ds^2=dt^2-a(t)^2(\mathrm{d}x^2+\mathrm{d}y^2+\mathrm{d}z^2)$$ for a spatially flat spacetime. We will also consider a power-law cosmology, such that $$ a(t)=a_{0}t^{s}$$, where *s* is a constant. Now using the nonlocal formalism, we can observe from Eq. (5)

12 $$\begin{aligned} \mathcal {G}[T]&=-\int _{t^{*}}^{t}\frac{\mathrm{d}t'}{e(t')}\int _{t^{*}}^{t'}\mathrm{d}t''e(t'')T(t''),\end{aligned}$$

13 $$\begin{aligned}&=\frac{6 s^2 }{(1-3 s)^2}\left[ 1-\left( \frac{t}{t^{*}}\right) ^{1-3 s}\right] -\frac{6 s^2 \log \left( \frac{t}{t^{*}}\right) }{3 s-1}. \end{aligned}$$

**Fig. 1:**
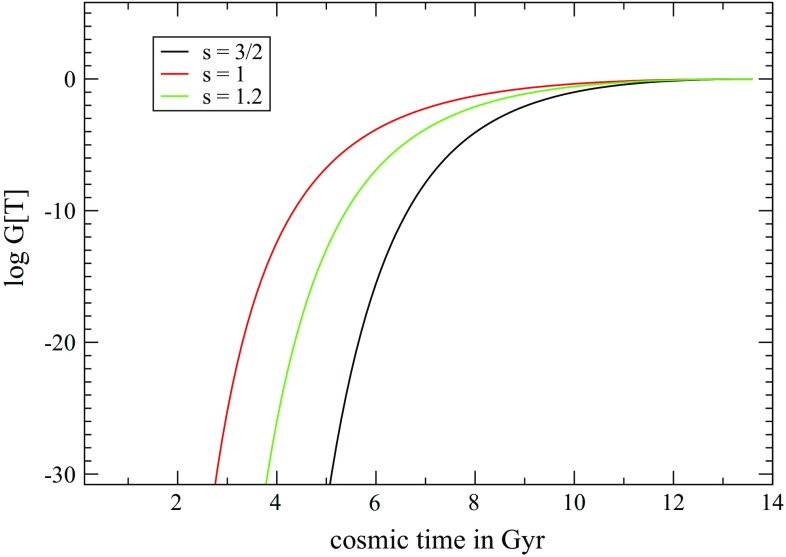
Evolution of $\mathcal {G}[T]$ as a function of the cosmic time in Gyr for some values of *s*

This can be used to analyze the effect of nonlocal deformation in teleparallel cosmology. From Fig. 1 we can observe the evolution of the function $\mathcal {G}[T]$ for the universe dominated by a certain form of matter ($s = 3/2$) and for the universe dominated by a specific scalar field (s = 1, 1.2).

## Observational constraints

In this section, we will analyze some observational constraints for nonlocal teleparallel cosmology. As discussed in [49], to analyze the observational constraints, we first express the nonlocal action in Eq. (3), in terms of two auxiliary scalar fields $\phi$ and $\xi$. In our case, we have

14 $$\begin{aligned} \mathcal {S}= & {} \frac{1}{2\kappa }\int \mathrm{d}^{4}x\, e \Big [T(f(\phi )-1)\nonumber \\&-\partial _{\mu }\xi \partial ^{\mu }\phi -\xi T\Big ]+\int \mathrm{d}^{4}x\, e\,L_{m}. \end{aligned}$$

By varying this action with respect to $\phi$ and $\xi$ we get $\phi =\square ^{-1}T$ and $\square \xi =-f'(\phi )T$ respectively. By varing this nonlocal action with respect to the tetrads, we obtain

15 $$\begin{aligned}&2(1-f(\phi )+\xi )\left[ e^{-1}\partial _\mu (e S_{a}{}^{\mu \beta })-E_{a}^{\lambda }T^{\rho }{}_{\mu \lambda }S_{\rho }{}^{\beta \mu }-\frac{1}{4}E^{\beta }_{a}T\right] \nonumber \\&\quad -\frac{1}{2}\Big [(\partial ^{\lambda }\xi )(\partial _{\lambda }\phi )E_{a}^{\beta }-(\partial ^{\beta }\xi )(\partial _{a}\phi )\nonumber \\&\quad -(\partial _{a}\xi )(\partial ^{\beta }\phi )\Big ] -2\partial _{\mu }(\xi -f(\phi ))E^\rho _a S_{\rho }{}^{\mu \nu }= \kappa \Theta ^\beta _a. \end{aligned}$$

Thus, the field equations can be written as

16 $$\begin{aligned} 3H^2(1+\xi -f(\phi ))=\frac{1}{2}\dot{\xi }\dot{\phi }+\kappa (\rho _{m} + \rho _{\Lambda }), \end{aligned}$$

17 $$\begin{aligned}&(1+\xi -f(\phi ))(3H^2+2\dot{H})=-\frac{1}{2}\dot{\xi }\dot{\phi }+2H(\dot{\xi }-\dot{f}(\phi ))\nonumber \\&\quad -\kappa (p_{m} + p_{\Lambda }), \end{aligned}$$

where dots represent differentiation with respect to the cosmic time and we have assume that the matter is described by the energy density of standard matter $\rho _m$ and an energy density related to a cosmological constant $\rho _{\Lambda }$. The equations for the scalar fields can be written as

18 $$\begin{aligned}&-6H^2 f'(\phi )+3H \dot{\xi }+\ddot{\xi }=0, \end{aligned}$$

19 $$\begin{aligned}&3H\dot{\phi }+6H^2+\ddot{\phi }=0. \end{aligned}$$

These equations describe a nonlocal model of teleparallel cosmology. We can take into account constraints on them from recent cosmological data. We will assume $f(\phi ) = A \exp (n \phi )$, in order to test the dynamics of the model given by the system (16)–(19). In order to constrain the free parameters of the model, we consider the following data sets:

**Fig. 2:**
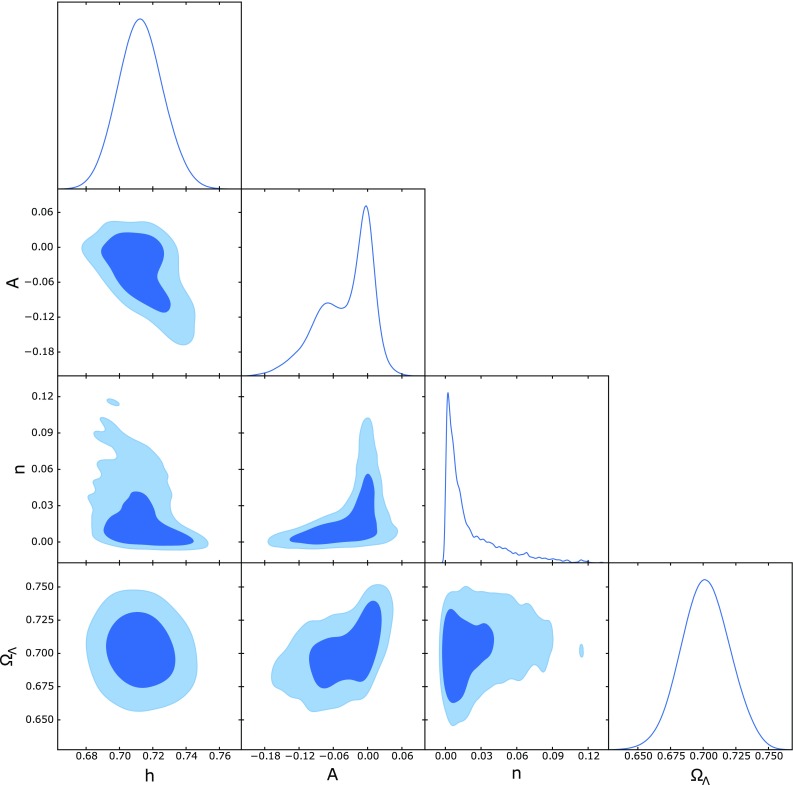
One-dimensional marginalized distribution, and two-dimensional contours with 68 and 95% confidence level for the free parameters of the model

**Fig. 3:**
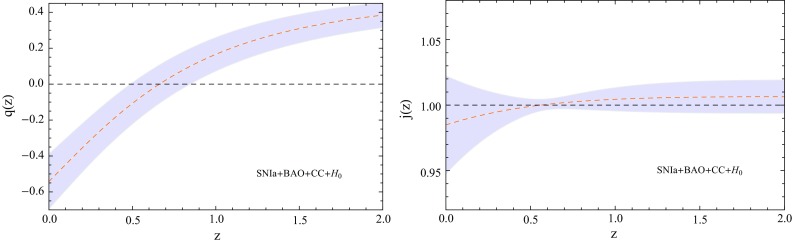
Reconstruction of the *q*(*z*) (deceleration parameter) and *j*(*z*) (jerk parameter) from $SNIa + BAO + CC + H_0$ data set at 1 $\sigma$ CL

**SNe Ia**: Type Ia supernovae (SNe Ia) have been used to discover the current stage of accelerated expansion of the universe. Hence, these observational data are a powerful tool for geometric tests. Here, let us adopt the latest “joint light curves” (JLA) sample [50], comprised of 740 type Ia supernovae in the redshift range $0.01 \le z \le 1.30$.

**BAO**: The baryon acoustic oscillations (BAOs) are another important probe. We use the BAO measurements from the Six Degree Field Galaxy Survey (6dF) [51], the Main Galaxy Sample of Data Release 7 of Sloan Digital Sky Survey (SDSS-MGS) [52], the LOWZ and CMASS galaxy samples of the Baryon Oscillation Spectroscopic Survey (BOSS-LOWZ and BOSS-CMASS, respectively) [53], and the distribution of the LymanForest in BOSS (BOSS-Ly) [54]. These data points are summarized in Table I of [55].

**CC+** $H_0$: The cosmic chronometers (CC) data set are another important data set. Here, we use the CC data set comprising 30 measurements spanned in the redshift range $0< z < 2$, recently compiled in [56]. We also use the recently measured new local value of Hubble constant given by $H_0= 73. 24 \pm 1.74$ km/s/Mpc.

We use the publicly available CLASS [57] and Monte Python [58] codes for the model under consideration in order to constrain the free parameters of this nonlocal cosmological model using SNe Ia + BAO + CC + $H_0$. We used the Metropolis Hastings algorithm with uniform prior on the model parameters. In our analysis, we considered $\ddot{\phi } \ll \dot{\phi }$, $\ddot{\xi } \ll \dot{\xi }$. Figure 2 shows the parametric space for *A*, *n*, $H_0$, and $\Omega _{\Lambda }$, at 1$\sigma$ and 2$\sigma$ confidence levels (CL) from the joint analysis SNIa + BAO + CC + $H_0$. We have observed at 1$\sigma$ CL the following constraints: $A=-0.009713_{-0.021}^{+0.017}$, $n=0.02086_{-0.0208}^{+0.0013}$, $h = 0.7127_{-0.015}^{+0.013}$ km/s/Mpc, $\Omega _{\Lambda }=0.7018_{-0.02}^{+0.018}$, and $\Omega _{m0}=0.2981_{-0.018}^{+0.02}$, with $\chi ^2_{min}=707.4$. We can note that the constraints are closed to the $\Lambda$CDM model, without any evidence for nonlocal effects in the present analysis, which here are characterized by the parameters *A* and *n*. In order to investigate kinematic effects, Fig. 3 shows the deceleration and jerk parameters as a function of the redshift. We consider the standard error propagation using the best fit values from SNIa + BAO + CC + $H_0$ in the reconstruction (gray region) of both parameters. On the left panel we have *q*(*z*), where the transition from decelerated to accelerated phase occurs at $z \sim 0.6$, with $q_0 = -0.54 \pm 0.15$. As expected, we have $q \rightarrow 1/2$ for high redshift. The right panel shows the jerk parameter *j*(*z*) obtained from the joint analysis, the dotted black line ($j = 1$) represents the $\Lambda$CDM model. In general, small deviations can be noted when nonlocal effects are introduced, but such effects are close the dynamics of $\Lambda$CDM model.

The free parameters of the nonlocal teleparallel cosmology are strongly constraint by present cosmological data. Furthermore, since nonlocal GR and nonlocal teleparallel gravity are fundamentally different, it is possible that future cosmological data can be used to test which of these two proposals is the correct theory of gravity.

As these theories are fundamentally different, experiments can be performed to distinguish each other. Here we propose some possible experimental tests that can be pursued in the near future to know which is the correct theory of gravity.

The first experiment that can be performed is based on the violation of the equivalence principle, as this can only occur in nonlocal teleparallel gravity. The accuracy of weak equivalence principle has been measured from the acceleration of beryllium and titanium test bodies using a rotating torsion balance [59]. It has been found that, for acceleration *a*, the accuracy is of the order $\Delta a /a \sim 1.8 ^{-13}$. The accuracy is increased to $\Delta a /a \sim 2 ^{-17}$ using the SR-POEM project [60]. It is possible to use more accurate future experiments to observe a violation of the weak equivalence principle. As such, a violation would only occur in nonlocal teleparallel gravity and it can be used as an experimental test to know which of these theories is the correct theory of nature.

We can also test these theories by performing experiments using photon time delay and gravitational red shifts measured by high energy gamma rays. Both these nonlocal effects would produce different photon time delays that have been observed by measuring the round trip time of a bounced radar beam off the surface of Venus [61]. This kind of experiments, performed with more precision, can be compared with effects produced by the nonlocal deformation of both theories, and any discrepancy between results can be used to discriminate between them. Similarly, the gravitational red shift can be used to distinguish between the two theories. The gravitational red shift derived by gamma rays of energy $14.4 \times 10^{-6}$ GeV has been measured in the Pound–Snider experiment [62], and it is possible to perform similar experiments with higher energy gamma rays with present day technology. Since Nonlocal teleparallel gravity and nonlocal GR predict different gravitational red sifting, such difference can be compared with these more accurate experiments.

## Conclusions

Since GR and TEGR produce the same classical field equations, they cannot be differentiated by using classical experiments. However, these theories are fundamentally different each other and so they have to produce different quantum mechanical effects. According to this consideration, GR and teleparallel gravity could be distinguished only at the quantum level. Even though we do not have a fully developed theory of quantum gravity, there are several proposals in this direction. A universal prediction by almost all the approaches is the existence of extended structures of spacetime geometry that are expected to give rise to nonlocal deformations whose effects could be detectable from microscopic scales to cosmology. Thus, the nonlocal deformations of teleparallel gravity, just like the nonlocal deformations of GR, are motivated by quantum gravitational effects. In principle, nonlocal cosmology from GR predicts a different behavior with respect to nonlocal teleparallel cosmology. Thus, the nonlocal deformations of these cosmological models can be matched with observational data. In analogy with a nonlocal deformation of GR, we constructed a nonlocal deformation of teleparallel gravity. Starting from this, we derived nonlocal cosmological solutions and constrained them using data coming from SNeIa, BAO, and CC surveys. The main result of this paper is that nonlocal teleparallel gravity is consistent with present cosmological data and then cosmology, besides quantum experiments, could be the ground on which to discriminate the two approaches. As a general consideration, nonlocal deformations for both GR and teleparallel gravity are different, and the parameters of the field equations can be fixed, in principle, by experiments. Here we proposed also future experiments that can be performed to distinguish them from each other.

## Appendix A: Derivation of the field equations

### 1. Variation of $f(\mathcal {G}[T])=f(\square ^{-1}T)$

Let us consider the variation of the action (3) with respect to the tetrad fields. The term with the quantity $f(\square ^{-1}T)$ is

A1 $$\begin{aligned} eT\delta f(\mathcal {G}[T])&=eT\delta f\Big (\square ^{-1}T\Big )\nonumber \\&=eTf'(\mathcal {G})\Big (\frac{1}{\Box }\delta T-\frac{1}{\Box } (\delta \Box ) \, \frac{1}{\Box }T\Big )\end{aligned}$$

A2 $$\begin{aligned}&=-e\Box ^{-1}(Tf'(\mathcal {G}))\delta \Big (\frac{\partial _{\mu }eg^{\mu \nu }\partial _{\nu }}{e} \Big )\Box ^{-1}T\nonumber \\&\quad +e\Box ^{-1}(Tf'(\mathcal {G}))\delta T. \end{aligned}$$

We will not work out the second term on the right hand side since the variation of $F(e)\delta T$ is well known for any function *F*(*e*) which depends on the tetrad. Now, if we expand the first term, we get

A3 $$\begin{aligned}&-e\Box ^{-1}(Tf'(\mathcal {G}))\delta \Big (\frac{\partial _{\mu }eg^{\mu \nu }\partial _{\nu }}{e} \Big )\Box ^{-1}T\nonumber \\&\quad =(\Box ^{-1}Tf'(\mathcal {G}))T\delta e-\Box ^{-1}(Tf'(\mathcal {G}))\partial _{\mu }\delta (eg^{\mu \nu }\partial _{\nu })\Box ^{-1}T,\end{aligned}$$

A4 $$\begin{aligned}&\quad =T\mathcal {G}[Tf'(\mathcal {G})]\delta e+\partial _{\mu }(\mathcal {G}[Tf'(\mathcal {G})])(\partial _{\nu }T)\Big (g^{\mu \nu }\delta e+e\delta g^{\mu \nu }\Big ), \end{aligned}$$

where we used $\Box \times \Box ^{-1}T=T$ and we neglected boundary terms. Now, if we take into account that $\delta e=e E_{a}^{\beta }e_{\beta }^{a}$ and $\delta g^{\sigma \rho }=-(g^{\sigma \beta }E^{\rho }_{a}+g^{\rho \beta }E^{\sigma }_{a})\delta e_{\beta }^{a}$ we can expand the above term yielding

A5 $$\begin{aligned}&-e\Box ^{-1}(Tf'(\mathcal {G}))\delta \Big (\frac{\partial _{\mu }eg^{\mu \nu }\partial _{\nu }}{e} \Big )\Box ^{-1}T\nonumber \\&\quad =e\Big [T\mathcal {G}[Tf'(\mathcal {G})]E_{a}^{\beta }+\partial _{\mu }(\mathcal {G}[Tf'(\mathcal {G})])(\partial _{\nu }T)\nonumber \\&\qquad \times \Big (g^{\mu \nu }E_{a}^{\beta }-2g^{\beta (\mu }E_{a}^{\nu )}\Big )\Big ]\delta e^{a}_{\beta }. \end{aligned}$$

Therefore, the variation of the nonlocal term is

A6 $$\begin{aligned} eT\delta f(\mathcal {G}[T])= & {} e\Big [T\mathcal {G}[Tf'(\mathcal {G})]E_{a}^{\beta }+\partial _{\mu }(\mathcal {G}[Tf'(\mathcal {G})])(\partial _{\nu }T)\nonumber \\&\times \Big (g^{\mu \nu }E_{a}^{\beta }-2g^{\beta (\mu }E_{a}^{\nu )}\Big )\Big ]\delta e^{a}_{\beta }\nonumber \\&+e\mathcal {G}[Tf'(\mathcal {G})]\delta T. \end{aligned}$$
